# Validation and Reproducibility of an App for Continuous Measurement as an Assessment Tool for Idiopathic Scoliosis

**DOI:** 10.3390/s26072099

**Published:** 2026-03-27

**Authors:** Isis Juliene Rodrigues Leite Navarro, Louis Jacob, Kevin Masetto, Francesco Dulio, Andrea Negrini, Stefano Negrini, Fabio Zaina, Alessandra Negrini

**Affiliations:** 1 ISICO (Italian Scientific Spine Institute), 20141 Milan, Italy; isis.navarro@isico.it (I.J.R.L.N.); kevin.masetto@isico.it (K.M.); francesco.dulio@isico.it (F.D.); andrew.negrini@gmail.com (A.N.); stefano.negrini@isico.it (S.N.); alessandra.negrini@isico.it (A.N.); 2 Department of Physical Medicine and Rehabilitation, Lariboisière-Fernand Widal Hospital, AP-HP, Université Paris Cité, 75010 Paris, France; louis.jacob@aphp.fr; 3 Epidemiology of Ageing and Neurodegenerative Diseases (EpiAgeing), Inserm U1153, Université Paris Cité, 75010 Paris, France; 4 Research and Development Unit, Parc Sanitari Sant Joan de Déu, CIBERSAM, ISCIII, Dr. Antoni Pujadas, 42, Sant Boi de Llobregat, 08830 Barcelona, Spain; 5 University of Milan, 20122 Milan, Italy; 6 IRCCS Galeazzi S.Ambrogio Hospital, 20161 Milan, Italy

**Keywords:** scoliosis, validation study, reliability study, clinical assessment, posture, mobile applications

## Abstract

**Highlights:**

**What are the main findings?**

- Continuous measurement of rib hump using a smartphone app during the Adams forward bend test shows very high concurrent validity and excellent intra- and interrater reliability compared to the scoliometer.
- Sagittal plane measurements (thoracic kyphosis and lumbar lordosis) obtained with the app demonstrate moderate reproducibility, with lower reliability for lumbar lordosis.

**What are the implications of the main findings?**

- Smartphone-based continuous ATR assessment can be used as a reliable, radiation-free clinical tool for monitoring transverse plane deformities in adolescents with spinal deformities.
- With appropriate examiner training, app-based sagittal profile measurements may support clinical follow-up, though caution is advised for lumbar lordosis evaluation

**Abstract:**

(1) Background: Idiopathic scoliosis is a three-dimensional deformity, yet clinical and research decision-making still relies largely on radiographic Cobb angle measurements. As a radiation-free alternative, clinical assessment of transverse and sagittal plane deformities has gained importance. This study evaluated the concurrent validity and intra- and interrater reproducibility of continuous measurements of rib hump, thoracic kyphosis, and lumbar lordosis obtained using a smartphone application in adolescents with spinal deformities. (2) Methods: Adolescents aged 10–17 years with scoliosis (>10° Cobb) or hyperkyphosis (>50° Cobb) were recruited. Continuous measurements of angle of trunk rotation (ATR) during the Adams forward bend test and in standing position, as well as sagittal profile, were collected using the ISICO app mounted on a standardized plastic tool. Concurrent validity was assessed against a scoliometer using Spearman correlation, root mean square error, and Bland–Altman analysis, while reproducibility was evaluated using intraclass correlation coefficients, standard error of measurement, and minimal detectable change. (3) Results: Thirty-two adolescents were included for validation and intrarater analyses and 34 for interrater analyses. ATR measured during the Adams test showed very high correlation with the scoliometer and minimal bias, while standing ATR showed moderate correlation. Reliability was excellent for rib hump during forward bending and moderate for sagittal parameters, with the lowest values observed for lumbar lordosis. (4) Conclusions: These findings support the clinical use of continuous app-based ATR assessment and suggest that sagittal measurements may be useful with appropriate examiner training.

## 1. Introduction

With an estimated global prevalence of nearly 2% of the world’s population [1], idiopathic scoliosis has been well defined in recent decades as a three-dimensional condition of the spine and trunk [2]; the Cobb angle [3] measured on the anteroposterior radiograph remains the main variable of clinical and scientific interest [4]. Similarly, sagittal plane alterations, such as hyperkyphosis, also rely on the Cobb angle measured in the sagittal plane as a guiding parameter for clinical decision-making. However, in recent years, there has been a growing effort among researchers and clinicians to develop accurate and reproducible methods of assessment, encompassing clinical variables across all three planes [5,6].

Clinical evaluation in the transverse and sagittal planes has gained relevance and is now widely used in the diagnosis and follow-up of patients with scoliosis [7]. Well known among specialists in the field, the measurement of the angle of trunk rotation using the scoliometer [8] provides a single-point measurement of trunk rotation, expressed as a discrete variable with an accuracy of 1° [9]. In the sagittal plane, the measurement of plumb-line distances, followed by the calculation of the sagittal index given in millimeters to estimate thoracic kyphosis, as well as the measurement through the surface as the flexicurve and the inclinometer, has been systematically adopted by professionals in the area [10,11,12,13].

Innovation in assessment tools, driven by technological advances, has led to the search for less invasive, more precise, accurate, and practical instruments and techniques. So far, mainly expensive tools have been developed and applied, like the surface topography, raster topography, and Moiré pattern. Despite their utility, there are limitations in their use in clinical practice, mainly due to the costs, their size and their lack of portability [14]. Within this context, the ISICO app was developed to enable continuous measurement of the rib hump not only in the classical forward-bending position, but also in the standing position, allowing for a better understanding of curve morphology, as well as continuous assessment of sagittal curves in standing posture.

In this scenario, the widespread availability of smartphones equipped with advanced embedded sensors offers a promising opportunity to bridge the technical and economic gap in the monitoring of health conditions. Similar to their increasing use in applications for dietary tracking, physical activity monitoring, and vital sign assessment through wearable technologies, smartphones have the potential to provide accessible, low-cost, and portable solutions for clinical evaluation. Their integration into healthcare enables the translation of complex measurement techniques into user-friendly tools that can be easily implemented in routine clinical practice, thereby expanding access to quantitative assessment beyond specialized settings.

This new assessment tool aims to determine the angular magnitude, extent, and morphology of the hump, and to provide measurements of thoracic kyphosis and lumbar lordosis in the sagittal plane. In addition, it allows for the extraction of measurements across different planes using a single instrument. Recognizing the importance of determining the measurement properties of new assessment tools, this study aims to validate and verify the reliability of continuous measurements in the transverse and sagittal planes obtained with the ISICO app in a sample of patients with spinal deformities during growth.

## 2. Materials and Methods

### 2.1. Study Design

This is a prospective, observational study designed to assess validation and reproducibility. The study report adheres to the GRRAS (Guidelines for Reporting Reliability and Agreement Studies) checklist for observational studies on reliability and agreement [15].

### 2.2. Participants

We included a convenience consecutive sample, consisting of patients of both sexes who were either already in treatment or attending their first medical consultation at a tertiary-level rehabilitation center specialized in spinal disorder treatment. We composed two completely independent samples, consisting of different patients: one sample for the validation and intrarater reproducibility analysis, and another sample for the interrater reproducibility analysis. Data collection for this study was conducted from April to June 2025 in Milan, Italy, and the data collection time for each participant was approximately 5 min.

Inclusion criteria were a diagnosis of vertebral deformity (scoliosis >10° Cobb or hyperkyphosis >50° Cobb) during growth, age between 10 and 17 years, and Risser stage 0–5. Participants were excluded if they had undergone spinal surgery, reported vertebral pain, or had a diagnosable cause of scoliosis (secondary or congenital scoliosis). Ethical approval was granted by the Research Ethics Committee of Lombardy 3 ID 3416 under protocol number 3416_SA_03.02.2025_P_bis. Written informed consent was obtained from the parents of all participants.

For sample size calculation, a null hypothesis of zero agreement using ICC was considered. Two measures per patient were tested; a minimum sample size of 22 patients was required for 80% power, with an expected ICC of 0.5 considered acceptable. For 90% power and a minimum expected reliability of 0.6, the required sample size was 20 patients. Considering routine clinical data collection and a low expected rate of missing data, a potential 25% loss due to system errors was estimated, rounding the sample to a minimum 25 prospectively recruited patients.

### 2.3. Data Collection and Signal Processing

The Continuous Measurement (CM) test implemented in the ISICO app (compatible with Android 7.0 or later [Google LLC, Mountain View, CA, USA], and iOS 16.6 or later [Apple Inc., Cupertino, CA, USA]) is designed to collect objective parameters during clinical assessment of spinal deformities. It supports the measurement of trunk asymmetry in the transverse plane (rib hump, assessed during the Adams forward-bending test using the Bunnell method; angle of trunk rotation—ATR) and of the sagittal profile (clinical measurement of thoracic kyphosis and lumbar lordosis).

**Figure 1:**
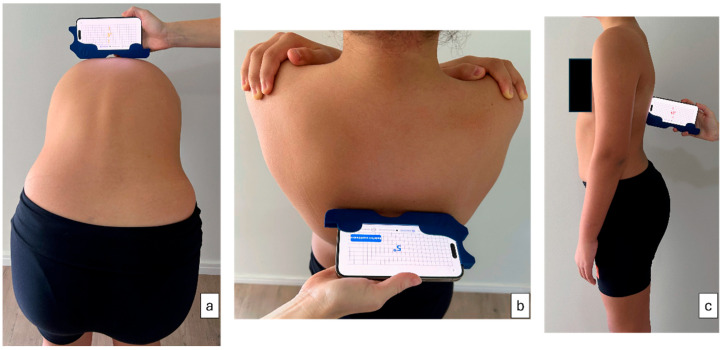
Use of the plastic tool attached to a smartphone for continuous measurement acquisition: (**a**) measurement of trunk rotation during the Adams forward bend test; (**b**) measurement of trunk rotation in the standing position; (**c**) measurement of kyphosis and lordosis in the standing position.

During acquisition, the smartphone is inserted into a custom-designed plastic holder that standardizes its position and handling. The tool allows the device to be used either horizontally, reproducing the geometry of a standard scoliometer for ATR measurement (Figure 1a,b), or vertically, similarly to a clinical inclinometer [16] for sagittal measurements (Figure 1c).

#### 2.3.1. Sensor Acquisition (Android and iOS)

The app uses the smartphone’s internal motion sensors (inertial measurement units, IMUs), including accelerometer- and gyroscope-based signals, to estimate orientation in real time. On Android devices, signals are acquired through the system sensor framework. Rib hump and sagittal inclinometry are obtained primarily from the accelerometer, providing a stable sampling rate on the order of tens of measurements per second. The relevant angle is computed mathematically from the sensor components and corrected using a calibration offset defined by the operator prior to measurement.

For transverse rotation (torsiometer), the app uses the device’s orientation signal derived from combined sensor data (sensor fusion of accelerometer and gyroscope). To ensure stable and comparable measurements across different smartphones, values are stored at a controlled effective rate (approximately 10 measurements per second).

On iOS devices, acquisition is handled through the CoreMotion framework (typical update interval 0.01 s). Angles are derived from device attitude (yaw for transverse rotation, pitch for sagittal inclinometry) and benefit from the system’s internal sensor fusion and stabilization algorithms, resulting in low jitter and high reproducibility.

#### 2.3.2. Data Standardization and Normalization

Because the smartphone is moved along the patient’s back at variable speed depending on the operator, the raw data cannot be directly compared across acquisitions. Therefore, all recordings undergo a standardized processing step aimed at producing a consistent representation independent of scanning speed.

The recorded angular sequence is converted into a fixed-length profile by resampling the signal and computing average values in consecutive segments (“chunk averaging”). For whole-spine scans, the profile is normalized to 400 points. This approach ensures that the final representation reflects the shape of the curve, rather than the speed of execution. Importantly, this spatial resampling, combined with the subsequent polynomial fitting, standardizes the outputs and effectively eliminates differences in raw sensor sampling rates and jitter profiles between Android and iOS devices, ensuring cross-platform consistency. No additional filtering is applied at this stage.

#### 2.3.3. Server-Side Curve Reconstruction and Clinical Parameter Extraction

Once the normalized dataset is transmitted to the server, a dedicated algorithm reconstructs a smooth curve from the 400-point profile and extracts clinical parameters. To suppress residual high-frequency noise and sensor jitter while preserving clinically meaningful morphology, the normalized curve is fitted using a 9th-degree polynomial regression. This model order was selected empirically to provide sufficient flexibility for reconstructing complex spinal profiles—including multiple curvature inversions—while avoiding excessive smoothing of relevant local peaks. In addition to improving curve readability, polynomial fitting provides a stable mathematical representation that supports reliable parameter extraction through derivative-based analysis.

The reconstructed curve is then analyzed to derive the main clinical outcomes:

##### Transverse Plane (ATR/Rib Hump)

In the transverse plane, the fitted curve is analyzed to identify the maximum trunk rotation, corresponding to the most prominent peak of the profile. For clinical reporting, the ATR value is expressed as an absolute magnitude and rounded to the nearest whole degree, consistent with standard scoliometer practice.

##### Sagittal Plane (Kyphosis and Lordosis)

In the sagittal plane, the reconstructed profile is analyzed to identify:

- The apices of thoracic kyphosis and lumbar lordosis (maximum curvature regions),
- The transition points between spinal segments.

Derivative-based analysis is used to support stable localization of these features. The final output includes both the magnitude of kyphosis/lordosis and an estimate of their position along the spine.

#### 2.3.4. Data Collection

**Figure 2:**
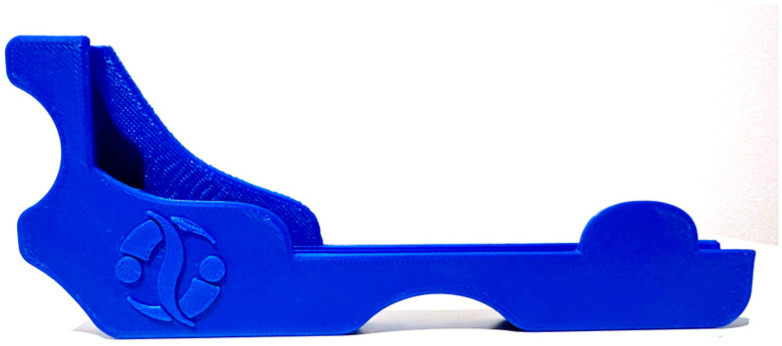
Plastic support tool to attach to a smartphone for continuous measurement acquisition.

For CM acquisition, all patients were assessed wearing minimal clothing with hair tied back. To ensure consistent positioning and orientation of the smartphone during acquisition, the device was mounted on a custom plastic support tool (Figure 2). This support standardizes contact with the patient’s back and minimizes variability related to hand positioning, contributing to measurement consistency across operators and devices. Patient positioning was standardized: they stood with the back exposed to the examiner, with feet approximately 20 cm apart. In forward bending with head flexed, arms relaxed, and legs extended (Adams Test position) [17], rib hump was measured with the ISICO app inclinometer using the Bunnell method. Vertebral rotation was recorded by sliding the instrument along the spine from C7 to S1 without applying pressure. For sagittal profile assessment, the patient stood in neutral posture; the instrument’s left side was placed on C7 and continuous measurement was recorded while sliding cranio-caudally to S1. Continuous measurement generates graphs for each variable, indicating slope points and reference segments for the sagittal profile.

The reliability of continuous measurement was determined by analyzing repeatability as well as intra- and interrater reproducibility. Three independent and blinded evaluators (E1, E2, E3) performed data collection simultaneously. The evaluators were physiotherapists with clinical experience in treating children with spinal deformities ranging from 6 to 15 years. All evaluators received standardized training lasting 30 min. To ensure the cross-platform validity of the ISICO app, data collection was deliberately performed using both Android and iOS smartphones. For intrarater reproducibility analysis, each patient was measured twice consecutively by E2. For interrater analysis, all three evaluators measured each patient once in each position. For the validation sample, after acquiring continuous measurements in both positions (Adams Test and standing posture), E2 additionally collected the angle of trunk rotation using a traditional scoliometer, which was adopted as the gold standard in this study.

#### 2.3.5. Data Analysis

All data processing was implemented in Python (version 3.11.5) using standard scientific libraries, and statistical analysis was performed in a blinded and independent manner using R software (version 4.4.2). Both procedures were performed by two researchers who were not involved in data collection. Variables obtained from continuous measurements were calculated as follows. The automatic computation of the metrics n1, n2, and n3 was applied exclusively to the sagittal standing position. Each numerical series derived from these observations and contained in the original files was analyzed using a peak-and-valley detection algorithm designed to identify the key turning points of the curve. The procedure was defined as follows:

– n2 was defined as the minimum (valley) point of the original series;
– n1 was defined as the first local peak to the left of the minimum (or the previous maximum if no peak was detected, or equal to n2 if n2 was located at the left edge);
– n3 was defined as the first local peak to the right of the minimum (or the subsequent maximum if no peak was detected, or equal to n2 if n2 was located at the right edge).

The identified values were integrated into the main database using the corresponding suffixes: n#_poli for nine-degree polynomial series and n#_orig for original series. The variables var1 and var2 were derived from the previously defined metrics (n1, n2, n3) according to the measurement acquisition position:

– For the forward bending position (Adams test) and rib hump in standing, values were directly assigned as var1 = n1_poli and var2 = n2_poli;
– For the sagittal standing position, variables were calculated by subtracting successive values, representing the relative amplitude between key points of the curve: var1 = n1_poli − n2_poli and var2 = n2_poli − n3_poli.

This derivation represents the pre- and post-minimum variations of the curve, respectively, providing a standardized and reproducible measure.

### 2.4. Statistical Analysis

Data were analyzed using the R software. For concurrent validity, correlation analysis (Spearman), root mean square error calculation, and Bland–Altman graphical analysis were performed. Correlation values were classified as insignificant (<0.3), low (0.3–<0.5), moderate (0.5–<0.7), high (0.7–<0.9), and very high (>0.9) [18].

Reproducibility was assessed using the intraclass correlation coefficient (ICC) with a two-way mixed model and absolute agreement (3, k). ICC values were classified as poor (<0.4), moderate (0.4–<0.75), or excellent (≥0.75). The standard error of measurement (SEM) was calculated as SEM = √(1 − ICC) × SD, and the minimal detectable change (MDC) was calculated as MDC = z × SD × √[2(1 − ICC)], both expressed in the unit of measurement tested, where z is the critical value corresponding to the desired significance level (α = 0.05).

## 3. Results

**Figure 3:**
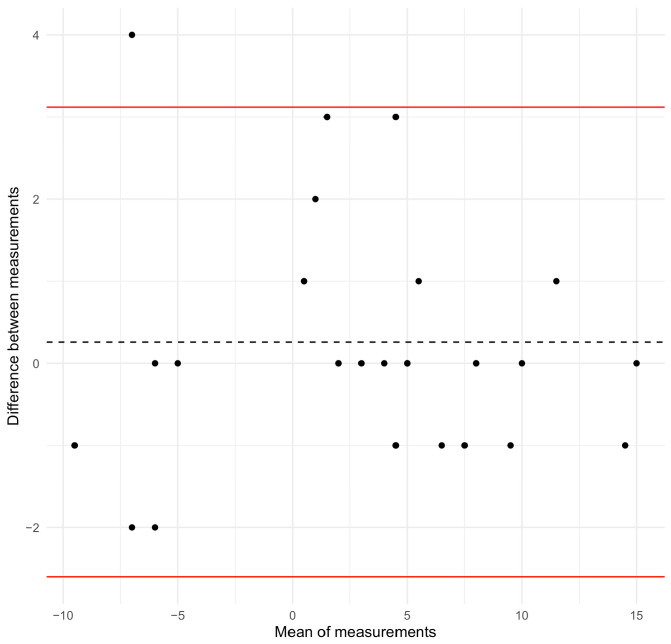
Bland–Altman plot for the ISICO-app rib hump measure versus the angle of trunk rotation while performing the Adams test. The red lines indicate the upper and lower limits. The dashed line indicates the bias.

For validation purposes, the sample was composed of 32 adolescents (Table 1). The median (interquartile range) age was 14.0 (3.3) years, with 71.9% of girls. The Spearman correlation coefficient was very right (ρ = 0.97; *p*-value < 0.001) between the angle of trunk rotation obtained through the continuous measurement using the ISICO app and the scoliometer, both acquired in forward bending position of the trunk (Adams test). After excluding angles of trunk rotation lower than 5° and 7°, based on clinical criteria for the diagnosis and treatment of scoliosis, the Spearman correlation coefficient was 0.97 and 0.99 (*p*-value < 0.001), respectively. The root mean square error was 1.46. The Bland–Altman plot of the relationship is displayed in Figure 3, with a bias of 0.26, and an upper and lower limit of −2.60 and 3.12 respectively.

**Figure 4:**
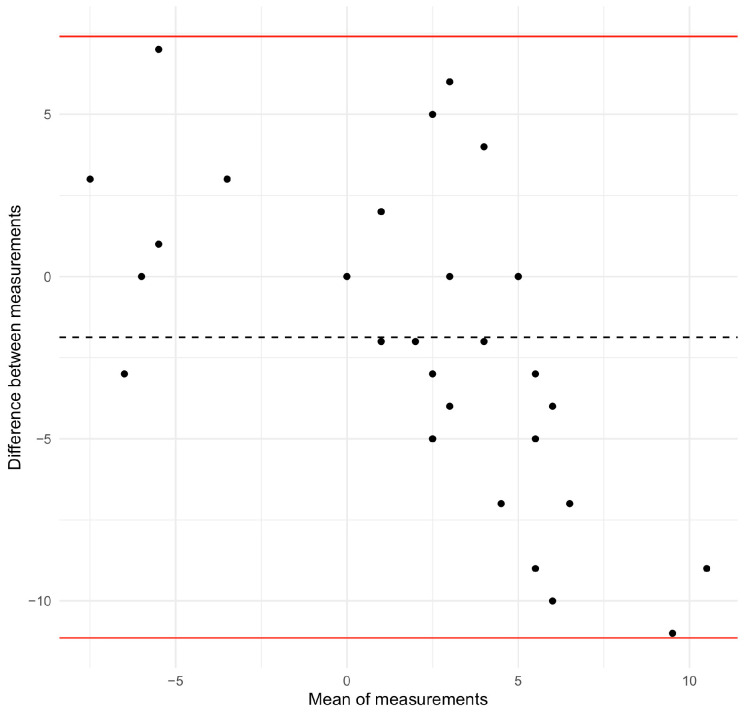
Bland–Altman plot for the ISICO-app rib hump measure versus the angle of trunk rotation while being in a standing position. The red lines indicate the upper and lower limits. The dashed line indicates the bias.

The relationship between these two variables was also studied when the participant was in a standing position. The Spearman correlation coefficient was 0.51 (*p*-value < 0.001), while the root mean square error was 5.02. When angles of trunk rotation lower than 5° and 7° were excluded, the Spearman correlation coefficient was 0.70 and 0.54 (*p*-value < 0.001 and 0.083) respectively. Finally, the Bland–Altman plot (upper limit −11.1; lower limit 7.4; bias −1.87) revealed that disagreement tended to increase between the ISICO-app measured rib hump and the angle of trunk rotation for lower values (Figure 4).

For the intrarater reliability of the ISICO app, the variables measured were rib hump (Adams Test and standing position), kyphosis and lordosis (in standing position). This analysis was conducted in the same sample as in the validation step. Table 2 shows the intrarater reliability of the ISICO app for the measured variables. The right rib hump measured during the Adams test displayed the highest intraclass correlation coefficient (0.93, 95% CI = 0.86, 0.97). This coefficient was the lowest for kyphosis in the standing position (0.54, 95% CI = −0.24, 0.75), see Table 3.

## 4. Discussion

Clinical assessment of patients with scoliosis is a priority recommendation among experts in the field, and the tools used to quantify imbalances across different planes must be reliable [6]. The present study demonstrated the validity of continuous measurement obtained using the ISICO app to assess the transverse plane, showing a very high correlation and low RMS error, as well as good agreement based on Bland–Altman graphical analysis. In addition, continuous measurement using the ISICO app showed moderate reliability for most sagittal plane measurements (thoracic kyphosis and lumbar lordosis) in both intra- and interrater analyses.

Considering that the period of highest scoliosis prevalence occurs during accelerated growth, typically between 10 and 14 years of age, avoiding repeated and excessive exposure to ionizing radiation is essential to promote overall patient health [19]. Within this context, numerous authors have sought to develop and validate new assessment tools and techniques for scoliosis that provide reliable, radiation-free measurements. To achieve this goal, clinical assessment has gained prominence, and surface measurements are frequently used to support clinical decision-making [20,21,22,23].

The use of the ISICO app for continuous measurement in the transverse plane offers an innovative approach to assessing trunk rotation both in the classical Adams forward bending test position and in the upright standing position. Other authors have also sought to measure trunk asymmetry using alternative instruments to the scoliometer and reported high correlations between the new tools and the gold standard (scoliometer). In a recent study using the Angle Pro smartphone application to measure the angle of trunk rotation (ATR), the authors found an excellent correlation between measurements obtained with the device using paper clips and those obtained with a scoliometer (r = 0.77 to 0.91) [24]. Similarly, another study using photogrammetry compared with a scoliometer reported high correlation values, corroborating our findings [9].

Despite being practical and low-cost alternatives, smartphone-based measurements using clips and photogrammetry allow trunk asymmetry assessment only in the classical Adams test position, and do not enable measurement in the upright position, as is possible with continuous measurement. The use of a smartphone with the ISICO app allows assessment in both positions (Adams test and standing). Measurement of trunk rotation in the upright position is usually explored using surface topography. In the study conducted in Italy by Aulisa et al., which comprised 95 patients, the authors reported ATR measurements both in the classical Adams test position and when standing to identify and quantify transverse plane deformities, with high correlation with clinical assessment when compared with surface topography measurements [25].

Still using surface topography for transverse plane assessment in patients with scoliosis, Mangone et al. compared vertebral rotation obtained with Formetric 4D in 25 patients with scoliosis, with the Raimondi angle obtained from radiographs. Significant but low correlations were found. An important point regarding our study is that the authors used the radiographic gold standard as the reference for comparison, which enhances the methodological quality of the study. Two limitations can be highlighted: first, measurements were performed only with patients in the Adams test position; second, the lack of comparison with clinical assessment, which could potentially yield better results and greater clinical applicability.

Furthermore, we would like to emphasize that continuous measurement of trunk rotation (rib hump), both in the classical Adams forward bending position and in standing posture, represents a novel approach for the assessment of patients with scoliosis. A key advantage of this method is the ability to generate a graphical representation of the rib hump profile, reflecting its overall shape. This allows for a more comprehensive evaluation, not only of its magnitude but, importantly, of its spatial extent.

In addition, future studies exploring these graphical outputs—particularly parameters such as the area under the curve—may significantly contribute to a deeper understanding of the deformity and to the evaluation of the effects of conservative treatment, with and without bracing.

In the present study, in addition to verifying the validity of continuous ATR measurement, we also tested its intra- and interrater reproducibility, as well as sagittal plane measurements of thoracic kyphosis and lumbar lordosis. ICC values for ATR measurements ranged from moderate to excellent (ICC = 0.56 to 0.93), with the highest reproducibility observed for right-sided trunk rotation in the intrarater analysis. High intra- and interrater ICC values were also reported by Nakaizumi et al. and Wei et al. [8,9] when testing ATR reproducibility using photogrammetry and surface scanning, respectively. Furthermore, in a study conducted with 51 patients using the Spinal Mouse to measure the Cobb angle in the frontal plane, the authors reported excellent ICC values in both intra- and inter-observer analyses (0.96 and 0.87). Although this measurement was performed in the frontal plane, the handling of the device and the examination procedure itself are similar to the continuous measurement performed in our study [26].

In the sagittal plane, the variables typically monitored in patients with scoliosis and other spinal conditions are the angular values of thoracic kyphosis and lumbar lordosis, traditionally obtained only through panoramic standing radiographs. New alternatives for measuring these variables have been described and tested by different authors, demonstrating high-to-very-high reproducibility [27]. Negrini et al. described and reported the validity of the sagittal index, a clinical measure easily obtained by measuring plumb line distances and subsequently calculating the index, which represents thoracic kyphosis on radiographs [28]. The authors also reported normal reference values for each measured distance and for the sagittal index, and defined the distance measured at L3 as representative of lumbar lordosis on radiographs [10].

Although highly useful in clinical practice and easy to apply, the repeatability of this method was reported as fair to good, corroborating our findings for intrarater thoracic kyphosis and interrater lumbar lordosis measurements. On the other hand, our results were superior for intrarater lumbar lordosis measurement. As this is a novel measurement technique, it may require greater professional training. Factors such as execution speed and maintenance of continuous skin contact may influence the quality of the obtained measurements.

In the study by Elpeze et al., the authors reported high ICC values for thoracic kyphosis measurements using both a flexicurve and a smartphone (0.96 and 0.94) in intra- and interrater analyses, respectively [29]. Although thoracic kyphosis was measured using a smartphone in a manner similar to our study, a fundamental difference lies in the static use of the smartphone, which was positioned at T1 for calibration and at T12 to obtain the angular value of thoracic kyphosis. The absence of movement during measurement may have contributed to the higher reproducibility observed when compared with our findings.

Continuous measurement using the ISICO app on a smartphone emerges as a novel quantitative approach for assessing the spine in both the transverse and sagittal planes, enabling measurements in both planes with a single instrument. Future studies exploring continuous measurement data in a more comprehensive and complex manner are needed to clarify its true utility in the assessment and monitoring of patients with scoliosis and other spinal conditions. In the present study, we intentionally adopted a basic analytical approach, as we considered it necessary to establish, in a simple and objective manner, the validity and reproducibility of widely known clinical measures, thereby paving the way for future innovative studies in this field.

To avoid restricting the use of the application to a single operating system, the data used for intrarater analysis were collected using two different smartphone models (Android and iOS), and reproducibility was confirmed for both systems.

Among the limitations of this study is the need for familiarization and training in app handling, as we hypothesize that this factor may influence the quality of the obtained measurements. In addition, the app does not currently provide immediate numerical outputs or graphical representations following continuous measurement. Furthermore, moderate reproducibility was observed for sagittal parameters, particularly lumbar lordosis; therefore, the use of the app for these variables should be interpreted with caution. Another potential limitation is the absence of comparison with radiographic data; however, we consider that surface-based clinical assessment was appropriately compared with the scoliometer, which can be regarded as the methodological gold standard due to its widespread clinical use and extensive scientific literature confirming its validity and reproducibility.

## 5. Conclusions

Continuous measurement using the ISICO app for ATR assessment is valid and reproducible, demonstrating high correlation values and low measurement error. In the sagittal plane, thoracic kyphosis and lumbar lordosis measurements obtained through continuous measurement with the ISICO app were reproducible, showing moderate coefficients across all analyses, with excellent reliability observed only for intrarater thoracic kyphosis measurement.

## Figures and Tables

**Table 1:** Characteristics of the populations included the study.

Characteristic	Category	First Population (*n* = 32) ^a^	Second Population (*n* = 34) ^b^
Age (in years)	Median (interquartile range)	14.0 (3.3)	13.0 (3.8)
Sex	Female	23 (71.9)	27 (79.4)
	Male	9 (28.1)	7 (20.6)
Weight (in kilograms)	Median (interquartile range)	52.2 (9.4)	50.0 (11.7)
Height (in centimeters)	Median (interquartile range)	164.0 (11.0)	164.0 (12.4)
Menarche	No	2 (8.7)	7 (25.9)
	Yes	21 (91.3)	20 (74.1)
Maximum Risser grade	0	5 (16.1)	7 (20.6)
	1	1 (3.2)	6 (17.6)
	2	7 (22.6)	6 (17.6)
	3	12 (38.7)	8 (23.5)
	4	3 (9.7)	6 (17.6)
	5	3 (9.7)	1 (2.9)

Data are *n* (%) unless otherwise specified. ^a^ The validation and the intrarater analyses were conducted in the first population. ^b^ The interrater analyses were conducted in the second population.

**Table 2:** Interrater reliability of the ISICO app for the measurement of the rib hump (Adams Test and standing position), kyphosis and lordosis (in standing position).

Measure	Position	Mean (SD)	ICC	95% CI	*p*-Value	SEM	MDC
Right	Adams test	4.8° (4.1°)	0.93	0.86, 0.97	<0.001	1.10	3.04
Left		−4.7° (4.0°)	0.56	0.25, 0.76	<0.001	2.64	7.31
Right	Standing position	2.6° (1.9°)	0.60	0.32, 0.79	<0.001	1.20	3.34
Left		−5.0° (3.0°)	0.69	0.45, 0.84	<0.001	1.70	4.72
Kyphosis	Standing position	30.5° (12.9°)	0.54	0.24, 0.75	<0.001	8.71	24.13
Lordosis		−43.2° (7.8°)	0.77	0.57, 0.88	<0.001	3.76	10.42

Abbreviations: SD, standard deviation; ICC, Intraclass correlation coefficient; CI, confidence interval; SEM, standard error of measurement; MDC, minimal detectable change.

**Table 3:** Interrater reliability of the ISICO app for the measurement of the rib hump (Adams Test and standing position), kyphosis and lordosis (in standing position).

Measure	Position	ICC	95% CI	*p*-Value	SEM	MDC
Right	Adams test	0.87	0.79, 0.93	<0.001	1.26	3.49
Left		0.87	0.78, 0.93	<0.001	1.29	3.57
Right	Standing position	0.68	0.50, 0.82	<0.001	2.28	6.32
Left		0.60	0.41, 0.75	<0.001	1.97	5.47
Kyphosis	Standing position	0.66	0.32, 0.83	<0.001	5.80	16.08
Lordosis		0.48	0.28, 0.67	<0.001	7.58	21.00

Abbreviations: ICC, Intraclass correlation coefficient; CI, confidence interval; SEM, standard error of measurement; MDC, minimal detectable change.

## Data Availability

The data supporting the findings of this study are available from the authors upon reasonable request, subject to applicable ethical and privacy considerations.
